# Editorial: Multidisciplinary approaches in pediatric gastrointestinal and liver diseases

**DOI:** 10.3389/fped.2025.1678818

**Published:** 2025-09-22

**Authors:** Maria Oana Săsăran, Ancuța Lupu, Vasile Valeriu Lupu

**Affiliations:** ^1^ Department of Pediatrics 3, George Emil Palade University of Medicine, Pharmacy, Sciences and Technology of Târgu Mureș, Târgu Mureș, Romania; ^2^ Department of Pediatrics, “Grigore T. Popa” University of Medicine and Pharmacy, Iasi, Romania

**Keywords:** pediatric gastrointestinal disorders, gastrointestinal dysmotility, hepatic and autoimmune diseases, acute gastroenteritis, children

**Editorial on the Research Topic**
Multidisciplinary approaches in pediatric gastrointestinal and liver disease

## 1 Introduction

Pediatric gastrointestinal diseases are complex and can frequently include extra-digestive manifestations, which can sometimes be markers of initial signs and symptoms. Hence, their diagnosis is often challenging, as the clinical picture might be misleading and could potentially point to a completely different condition. The research topic “Multidisciplinary approaches in pediatric gastrointestinal and liver diseases” aimed to attract publications focusing on the involvement of different disciplines in the diagnosis and management of pediatric gastrointestinal and hepatic disorders. A total of 10 articles were published under the umbrella of this research topic: six original research articles, two case reports, and two review articles.

## 2 Artificial intelligence application in pediatric gastrointestinal disorders

In recent years, artificial intelligence has boomed spectacularly, and ongoing studies suggest that it could be useful in the early diagnosis and prediction of complications of various pediatric pathologies. These include necrotizing enterocolitis in newborns, for which Cui et al. have proposed a multimodal, artificial intelligence-based model, based on standard laboratory investigations and abdominal x-ray. The authors’ unique combination of the multimodal model with the gradient-weighted class activation mapping (GradCAM) helps identify intestinal wall edema and gas, portal vein gas, and the dilation of the intestinal loops, improving the early recognition of necrotizing enterocolitis.

## 3 Gastrointestinal dysmotility conditions and pediatric bowel obstruction

Idiopathic constipation represents a problematic occurrence in children with neurological disorders, secondary to reduced intestinal motility. Within their study, Wang et al. analyzed the efficacy of Tui Na, a special massage therapy derived from Traditional Chinese Medicine, compared to that of oral lactulose in children with cerebral palsy, as provided in addition to basic treatment. The study proved that Tui Na performs similarly to traditional therapies such as lactulose in combating cerebral palsy-related constipation, proposing it is a viable alternative therapeutic approach. Addressing gastrointestinal dysmotility issues in children with psycho-motor delay can also involve therapies such as acetylcholinesterase inhibitors. Comisi et al. present a case report of a patient known with ATR-X syndrome, as well as gastro-esophageal reflux, dysphagia, and chronic constipation, in whom the barium enema highlighted a sub-stenosis of the recto-sigmoidian region. Pyridostigmine alleviated the abdominal pain and distention and improved the intestinal transit; thus, the need for surgery was avoided.

Foreign body ingestion is a concerning event in pediatrics, as toys made of components such as superabsorbent polymer (SAP) beads can lead to bowel obstruction. In an *in vitro* study, Hachem et al. assess the expansion of SAP in different liquid media, showing that gastric acid inhibits their expansion to a greater extent than water or alkaline solution. After administering solutions with high concentrations of polyethylene glycol (PEG), the size of the beads was effectively reduced, which makes PEG a prospective option in managing the ingestion of SAP beads in children.

Congenital or acquired bowel obstructions represent surgical emergencies that need to be recognized promptly and treated accordingly. Within a multicenter, retrospective study, Yang et al. point out that non-bilious vomiting is a frequent initial presentation in infants with intestinal malrotation. Still, abdominal ultrasound presents good sensitivity and specificity for the detection of intestinal malrotation complicated by intestinal obstruction, which makes further contrast examinations unnecessary. Acquired intestinal obstructions are uncommon complications of otherwise conservatively manageable conditions, such as eosinophilic gastrointestinal disease. Di Mari et al. report an unusual case of a male teenager with progressive duodenal stenosis, secondary to eosinophilic esophagitis and enteritis.

## 4 Hepatic and autoimmune diseases of the gastrointestinal tract

Hepatic congenital and autoimmune disorders have been thoroughly studied, but their diagnosis and outcome prediction still require further research, mainly based on the discovery of novel biomarkers. For example, activin A immunostaining of hepatic biopsy specimens, during hepatic portoenterostomy (HPE) performed for biliary atresia, has been proposed as a long-term outcome predictor of the surgical intervention. As Džepina et al. show in their research conducted on a group of infants with biliary atresia, the patients with the weakest positive reaction for activin A presented the highest survival rates, of over 90% 2 years after HPE, following native liver transplantation. The distinction of autoimmune hepatitis (AIH) from other AIH-like conditions, such as drug-induced hepatitis and infectious hepatitis, requires a thorough assessment of hepatic infectious antigens and antibodies, a hepatic antibody panel, as well as liver biopsy in some cases. Within a retrospective study, Ma et al. demonstrate that the identification of positive autoantibodies can aid in the early diagnosis of AIH. Furthermore, liver histology in AIH differs from other hepatic conditions through peculiar inflammatory modifications of the hepatic lobule, with lymphoplasmacytic infiltration.

Celiac disease is another condition that mainly leads to gastrointestinal symptoms and a malabsorption syndrome, but it behaves as a systemic disease, which can be associated with other immune-mediated diseases. Starcea et al. bring to attention the potential association between celiac disease and IgA nephropathy, as well as diabetic nephropathy, which both can lead to the development of chronic kidney disease, even at pediatric ages. Furthermore, a strong association exists between celiac disease and hyperoxaluria, a major risk factor for urolithiasis. These reported co-occurrences of renal disease in children call for periodic screening of renal function in pediatric patients with celiac disease.

## 5 Acute gastroenteritis in children

Acute gastroenteritis still remains a major problem in underdeveloped countries, accounting for one of the leading causes of mortality in children under the age of 5 years. Rotavirus is a major etiological factor of acute gastroenteritis outbreaks in both rural areas and hospital settings. Tian et al. have systematically reviewed the rotavirus outbreaks so far reported within the medical literature and concluded that group B rotaviruses lead to more severe outbreaks, characterized by a longer duration and a larger number of cases when compared to group A rotaviruses.

## 6 Conclusions

The research topic presented research articles on various topics; together, this outlines the importance of the implications of multiple domains and medical fields for the appropriate management and early recognition of pediatric gastrointestinal disorders. These included *in vitro* studies, artificial intelligence-based models, and studies/case reports focusing on surgical management or on the involvement of histology and immunostaining for the differential diagnosis and outcome prediction of hepatic conditions. Literature reviews of celiac disease-related renal conditions and rotavirus outbreaks in children complete the content of this research topic, showcasing the systemic and potentially fatal involvement of these conditions.

